# Virtual Reality in Clinical Teaching and Diagnostics for Liver Surgery: Prospective Cohort Study

**DOI:** 10.2196/60383

**Published:** 2024-11-27

**Authors:** Joshua Preibisch, Navid Tabriz, Maximilian Kaluschke, Dirk Weyhe, Verena Uslar

**Affiliations:** 1 General and Visceral Surgery, Pius Hospital Oldenburg, Oldenburg, Germany; 2 Computer Graphics and Virtual Reality, University of Bremen, Bremen, Germany

**Keywords:** VR, virtual reality, multiple-choice test, anatomy, patient-specific 3D visualization, MRI-based segmentation, liver, cohort study, visualization, tool, medical diagnostic, questionnaire, usability, diagnostics, surgery, 3D, MRI, magnetic resonance imaging

## Abstract

**Background:**

Learning and applying anatomy are essential but are studied and done through 2D tools and imaging techniques. This study aims to verify the usefulness of an additional 3D technique and ensure an improvement in the visualization of anatomical structures and pathological findings.

**Objective:**

The study aims to examine the usefulness of virtual reality (VR) technology as an additional tool in medical diagnostics. Groups of students, residents, and specialists in surgery, radiology, and internal medicine evaluated magnetic resonance imaging (MRI) by answering a multiple-choice questionnaire. Subsequently, a virtual 3D display was used for processing. The questionnaire focused on topographical conditions and the transfer of academic knowledge into clinical application. The main objective was to determine anatomical understanding in a comparison between sectional image (2D) presentation and additional VR (3D) presentation, measured through correctly answered questions and processing time. The system usability scale (SUS) was integrated as another criterion for VR usability.

**Methods:**

The cross-over study assessed 63 participants regarding their knowledge of liver anatomy and pathologies based on an interindividual comparison. Group formation according to the respective level of medical training was as follows: students (n=35), residents (n=15), and specialists (n=13). Participants answered 25 multiple-choice questions first using sectional imaging (MRI) in a 2D environment (computer screen) and afterward with the respective segmented 3D model visualized in a VR simulation. The main criteria for the analyses were the number of correctly answered questions and processing time. A customized SUS was used to analyze VR usability. Missing data analysis showed that there were no accounted missing data.

**Results:**

The rate of correct answers improved significantly with the additional use of VR (*F*_1,59_=314.376; *P*<.001). Using MRI, a significant difference was observed between students and residents (*P*=.04) and between students and specialists (*P*<.001). In the VR condition, no significant differences between groups were found. In the MRI condition, significant differences in processing time were observed between students and specialists (*P*=.02) and between residents and specialists (*P*=.04). No differences existed between students and residents. With VR, processing time decreased significantly in all groups (*F*_1,59_=280.700; *P*<.001). Significant differences between students and specialists (*P*=.02) and between students and residents (*P*=.004) remained. No notable differences between residents and specialists (*P*=.72) were found. The SUS showed a subjectively simplified answerability of the questions with additional use of VR. The usefulness and benefits for an additional use of VR were stated.

**Conclusions:**

The additional use of VR suggests statistically significant improvements across all groups. VR seems to enable students and residents to participate in diagnostics and create treatment plans at an early stage. Transferred to clinical practice, this may lead to improvement in diagnostics and interventions. The lack of randomization and a potential learning effect are the main limitations to be addressed in future studies.

## Introduction

### Background

Medical students invest significant time and effort in learning theoretical human anatomy. Usually, students have the classical 2D-anatomy atlases as well as lectures at the university as their primary learning method. An additional concept at medical schools is learning anatomy and especially topographical understanding through dissection courses or by working with preprepared cadaver parts as learning material. The transfer of theoretical knowledge to an activity, in this case, dissection, supports the learning process in the context of “learning by doing” [1]. Following the university education, a further learning process takes place within the framework of specialized further education. Especially in areas of surgery and radiology, but also for any medical specialty, an extraordinary level of knowledge of human anatomy, as well as the transfer of what has been learned to the respective patient, is indispensable for the success of diagnostics and therapy [2-4]. Sectional imaging techniques such as computed tomography (CT)/magnetic resonance imaging (MRI) used in a 2D environment (computer screen) are emerging in clinical practice as a basic tool for preparing and planning surgery and interventions. Here, among other things, guidelines prescribe the use of this conservative imaging for the evaluation of disease stages and indication for further therapies (staging) [5]. However, students and residents often find this challenging. Working with conservative imaging modalities for diagnostic purposes, such as MRI, requires good anatomical knowledge and a strong ability to transfer what is learned to a sectional view. Furthermore, the mental transition to a 3D picture is essential for the correct interpretation of the sectional image and further for the application of the gained information for practical interventions. In these different stages of applying what has been learned, the necessary change of dimensions can lead to significant problems [6,7]. In the theoretical framework of the cognitive load theory, a limit of working memory is stated, which affects learning and skill acquisition in correlation with the complexity of what is being learned. The difficulty of transferring what has been learned into a clinical setting can be cognitively overwhelming [8-10]. Considering the increasing technologization, gamification of learning tools is being worked on worldwide. The first studies regarding improved learning success with the help of computer games were already conducted in the 1980s [11]. 3D computer games motivate, are enticing, and convey 3D aspects well [6,8]. In the medical field, the use of a virtual 3D atlas (virtual reality [VR] atlas) for learning human anatomy is particularly interesting. Worthy of mention is the work of Höhne et al [12-14], who created 3D models for educational reasons from CT scans. Studies have shown that students who used a 3D atlas had better 3D visualization and anatomical-functional understanding [6,15,16]. In addition, learning with VR atlases increases learning satisfaction as well as efficiency and effectiveness, and VR-assisted learning is considered a useful adjunct to conventional anatomy instruction in dissection courses [17-19] and can reduce the cognitive load and improve learning success [8,20,21]. Taking this further, patient-specific datasets, for example, from conservative MRI can be segmented and transformed into 3D form, which can be examined in VR [22].

### Objectives

The objective of this paper is to examine the usefulness of VR technology as an additional tool in medical diagnostics. In this study, students, residents, and specialists in general and visceral surgery, radiology, and internal medicine evaluated magnetic resonance images by answering a multiple-choice (MC)–based questionnaire. Subsequently, a virtual 3D display was used for processing. The questionnaire was evaluated in terms of topographical understanding and the transfer of knowledge from university or the advanced training to clinical aspects. As analysis criteria, we used the number of correctly answered questions and the processing time. We included the system usability scale (SUS) as another criterion for the usability of VR [23,24]. The main objective was to check the anatomical understanding in a comparison between a 2D presentation and an additional VR (3D) presentation, measured by the number of correctly answered questions and response time based on the MC questions.

## Methods

### Study Design and Procedure

#### Study Design

The study design was based on an interindividual comparison in the form of a cross-over design. The experiment was conducted in 2 parts, which were performed after each other, within a time frame of approximately 1 hour. In both parts of the experimental design, a total of 25 MC questions were answered.

**Figure 1.**
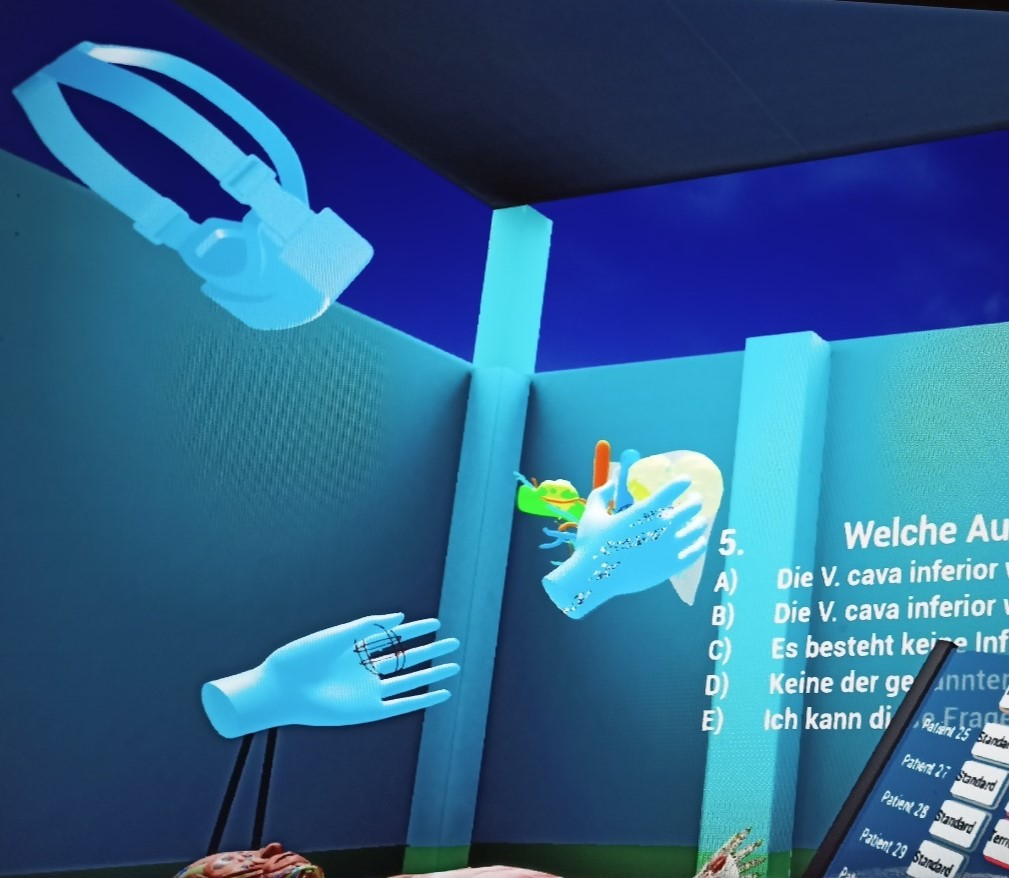
Exterior view from the study director on a person in the virtual reality environment; virtual representation of the operating room, participant and sequenced liver, digital menu for case selection, and questions projected on the wall.

In the first part, the participants were provided with the sectional imaging of an MRI presented on a 2D computer screen to answer the mentioned 25 MC questions. In the second part, we presented the respective segmented 3D model of the liver converted from MRI data and visualized in a VR simulation with VR goggles (HTC Vive Pro). In our experimental setup, participants found themselves in a simulated operating room with a virtual representation of a body on the operating table (Figure 1).

Controllers, which were held in both hands, allowed objects to be lifted and moved. During the inspection of the described liver anatomy, the virtual image of the liver appeared, floating in the operating room. A menu in the VR itself could be used to switch between the individual case data. Questions to be answered appeared on the wall of the operating room and were controlled externally to the VR scene by the study director. Questions were answered verbally and were noted on paper by the study director. Regarding time manageability, a maximum time of 3.5 minutes was provided per patient case, including 5 questions in each setting. We adhered to the general rules of the Institute for Medical and Pharmaceutical Examination Questions in developing the answer modalities, ensuring that, per question, only 1 answer option was classified as correct. Double negations were not used. Other types of questions were not used in the questionnaire. Questions that remained unanswered due to the time limit were marked as “wrong.” There was also the option to rate individual questions as “unanswerable,” this answer was also rated as “wrong” in the analysis.

Eventually, an adapted version of the SUS with 6 questions answerable on a 7-point Likert scale was answered by the participants, as well as 6 questions regarding the general benefits of the VR and MRI conditions [23,24].

#### VR Environment

The development and the design of the atlas itself were already described by Gloy et al [16]. This version of the anatomy atlas encompassed various features for the interaction with the anatomical dummy (see the walk-through video in Multimedia Appendix 1). Since then, the application has been further developed and expanded to include patient-specific 3D liver models that follow the mentioned interaction rules and the quiz described earlier.

The internal anatomy (tumors, blood vessels, and parts of the biliary tract system) of the patient-specific liver models was visualized with realistic lighting and unique opaque solid colors. On the other hand, the liver surface was visualized with back-to-front transparency, with a low opacity of 20%, where the light shade was also determined by the same realistic lighting model. In the case of the segmented view, the system rendered the liver surface with unique solid colors that corresponded to the segment, with the same opacity level as mentioned earlier. We iteratively tuned the rendering parameters to find the best compromise between making the liver contour, individual segments, and internal anatomy clearly visible.

The time required to create each model was approximately 1 day per model. A semiautomated process was used, where an algorithm recognized the structures of the reference MRI and converted them into a 3D model. The accuracy of the recognized structures was ensured by a radiological technical assistant. Thanks to the use of advanced software, it is now possible to segment a model in just 15 minutes.

VR was chosen in this study due to a high grade of immersiveness and interactivity as well as immersion. This provides an extraordinary perception of depth, which is lost by simply showing a 2D image or a 3D image viewed on a simple computer screen. Due to the external development of the VR software, there will be no distribution of the VR atlas.

#### Selection of Participants

When planning the number of participants, we decided on 40 students for pragmatic reasons. Further, a possible recruitment of 20 physicians was assumed. On the one hand, the planned number aligns with the usual cohort size for such user-centered studies. On the other hand, the recruitment of 60 study participants is considered realistic. Furthermore, there are few reliable data on the use of VR in anatomy teaching as well as in clinical applications, so reliable case number planning was not possible. During the study, preliminary analyses already showed clear results among the students, making further recruitment unnecessary.

Participants were all located within the health care system of Germany. As an overview of medical training in Germany, it should be mentioned that the degree program lasts 6 years of university studies, with students gaining their first clinical experience from the third year onward. Specialist training programs vary in length, typically lasting between 5 and 6 years. The study participants were selected so that each group was represented. The first group consisted of medical students in their fourth to sixth year. Residents from the departments of general and visceral surgery, internal medicine, and radiology formed the second group, and the third group was composed of specialists from the fields of general and visceral surgery, internal medicine, and radiology. Informed consent was obtained from study participants individually prior to initiation of testing.

#### Selection of Cases

We used specific medical cases for our study, focusing on metastases in the liver. The only inclusion criterion was the presence of 1 or more liver metastases as well as having undergone an MRI scan of the liver in preparation for surgery or further diagnostics. These MRI scans served as the basis for the segmentation and development of the 3D image. Case selection was performed retrospectively, in cooperation with the departments of general and visceral surgery and radiology, resulting in a total of 5 cases. Five MC questions were created for each of the 5 individual patient cases, resulting in a questionnaire of 25 MC questions. The questions developed were related to the present anatomy and topographical features of the patient’s liver (Multimedia Appendix 2). Per case 2, MRI sequences were selected to use for answering the questionnaire. These sequences contained T1-weighting as well as T2-weighting in 4 of 5 cases. One case contained only T1-weighting but with 2 different sequences.

#### Statistics

The descriptive analysis included the calculation of means, medians, and SDs related to participant characteristics, calculation of correct answers, and overall processing time per participant and condition. To test the data for normal distribution, the Shapiro-Wilke test was used. The normally distributed numbers of correctly answered questions were analyzed with a 2-way repeated measures ANOVA to show statistical differences. We used the method of imaging (MRI or VR) and the status of the participant (student, resident, or specialist) as the dependent variables. The processing time was defined as the total time required to answer the questionnaire. To calculate the total processing time, the time needed to answer all questions of the questionnaire regarding all 5 cases was added. The processing time data also were analyzed with a 2-way repeated measures ANOVA. We performed a missing data analysis, which showed that there were no missing data to account for.

For the data of the adapted SUS and the 6 questions pertaining to the general benefit of the VR system, the modus and the minimal and maximal values were calculated. All statistical tests and graphics were performed and created with SPSS Statistics (version 28.0.1.0; IBM Corp). All raw data are available in Multimedia Appendix 3.

### Ethical Considerations

The medical ethics committee of Carl von Ossietzky University accepted this study (application 2021‐162). The study was registered with the German Register of Clinical Studies (DRKS). Furthermore, we have committed ourselves to act according to the guidelines of the “Declaration of Helsinki” regarding the ethical principles for medical research on humans during the study. All participants signed a declaration of consent regarding participation. We followed the STROBE (Strengthening the Reporting of Observational Studies in Epidemiology) reporting guidelines when preparing the paper (Checklist 1). The selected image materials originated from patients of the Pius Hospital Oldenburg; the permission was obtained in the context of the consent of the patients. Participation of the participants was voluntary without given compensation. Before inclusion in the study, participants received detailed information about the study. Participants could withdraw from the study at any time without providing a reason and without facing any disadvantages. In the event of withdrawal from the study, data already obtained would either be destroyed or included in the study after inquiry as to whether the person agrees to the data being analyzed. This meant that the study participants had the right to have their data deleted if they withdrew from the study. The general abovementioned conditions regarding the handling of the collected data applied. All data were anonymized.

## Results

### Participant Characteristics

A total of 63 participants participated in our study. The participants were divided into 3 different groups. We categorized the participants as students, residents, and specialists of the departments of surgery, internal medicine, and radiology. In detail, we included 8 resident surgeons, 6 residents from internal medicine, 1 resident of the department of radiology. In the group of specialists, we included 10 surgeons, 2 specialists of the department of internal medicine, and 1 specialist of the department of radiology. In total, we included 34 students (Multimedia Appendix 4).

In percentage terms, the proportion of female participants is higher in the student group than in the other defined groups (students: n=25, 71%; residents: n=8, 53%; and specialists: n=2, 15%). There is also a wide range in age as well as years of experience, especially in the specialist group (mean age 45.6, SD 9.18; range 33-61 years; Table 1).

**Table 1.** Participant characteristics.

		Students	Residents	Specialists	Total
Participants (n)		35 (34^a^)	15	13	63 (62^b^)
**Age (years)**					
	Mean (SD)	26.8 (2.74)	30 (2.81)	45.6 (9.18)	—^c^
	Range	23-32	27-37	33-61	—
**Sex, n (%)**					
	Female	25 (71)	8 (53)	2 (15)	35 (55)
	Male	10 (28)	7 (46)	11 (84)	28 (44)
**VR^d^ experience, n (%)**					
	Never	24 (69)	6 (40)	7 (54)	37 (59)
	≤5 times	11 (32)	9 (60)	3 (23)	23 (37)
	5‐10 times	0 (0)	0 (0)	1 (8)	1 (2)
	>10 times	0 (0)	0 (0)	2 (15)	2 (3)
VR sickness, n (%)		1 (3)	0 (0)	0 (0)	1 (2)
**Experience (years)**					
	Mean (SD)	4.4	2.6	11.2	—
	Range	4-5^e^	1-5^e^	1-27^a^	—

a Included number of students.

b Included number of participants.

c Not available.

d VR: virtual reality.

e Years of experience regarding the group category.

**Figure 2.**
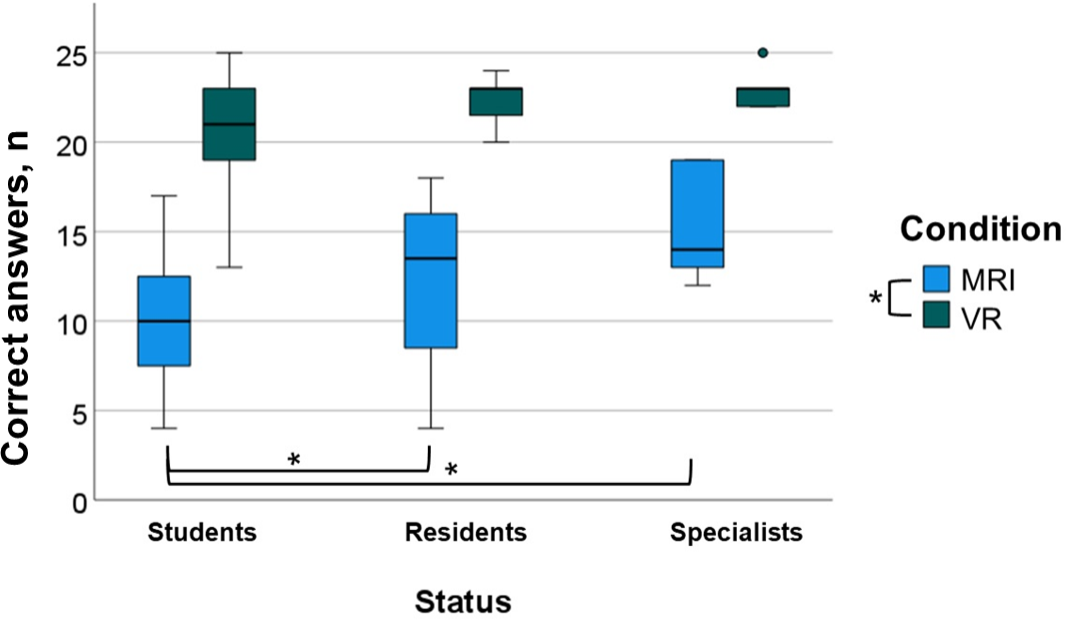
Number of correct answers; comparing the different conditions (blue=MRI and green=VR) and contrasting the status (student, resident, and specialist). The box plot shows the median, the 2 quartiles, and the extreme values. The black circled dot indicates an outlier value. MRI: magnetic resonance imaging; VR: virtual reality. *indicates statistical significance between the groups.

There was also a wide range of experience of using VR. Overall, 24 students indicated they had never used VR, and 11 students indicated they had used VR 1‐5 times. In the resident group, 6 participants had no VR experience, and 9 participants had used VR 1‐5 times. A total of 7 specialists reported never having used VR, 3 specialists used VR 1‐5 times, 1 noted use of VR 5‐10 times, and 2 participants had used VR more than 10 times (Table 1). During the study, 1 student participant was excluded from the study due to VR sickness (Table 1). The mean of all participants showed an average of 11.8 (SD 4.19) correctly answered questions using MRI and 21.8 (SD 2.44) correct answers with additional use of VR technology (n=25; Figure 2).

The multifactorial 2-way repeated measures ANOVA showed a significant difference in error rates between the MRI and the VR condition (*F*_1,59_=314.376; *P*<.001) and an effect size of 0.842. Further, a significant interaction between condition and status could be presented (*P*=.04). In the pairwise comparison analysis performed for the MRI condition only, a significant difference was found between students and residents (students: median 10, IQR 7.25-12.75 and residents: median 13, IQR 10-16; *P*=.04) as well as between students and specialists (students: median 10 IQR 7.25-12.75 and specialists: median 14, IQR 13-18; *P*<.001). However, there were no significant differences between residents and specialists (residents: median 13, IQR 10-16 and specialists: median 14, IQR 13-18; *P*=.18). For the VR condition, no significant differences between the sample groups can be shown (students: median 21, IQR 19.25-23; residents: median 23, 21.5-23; and specialists: median 23, IQR 22-23).

### Processing Time

**Figure 3.**
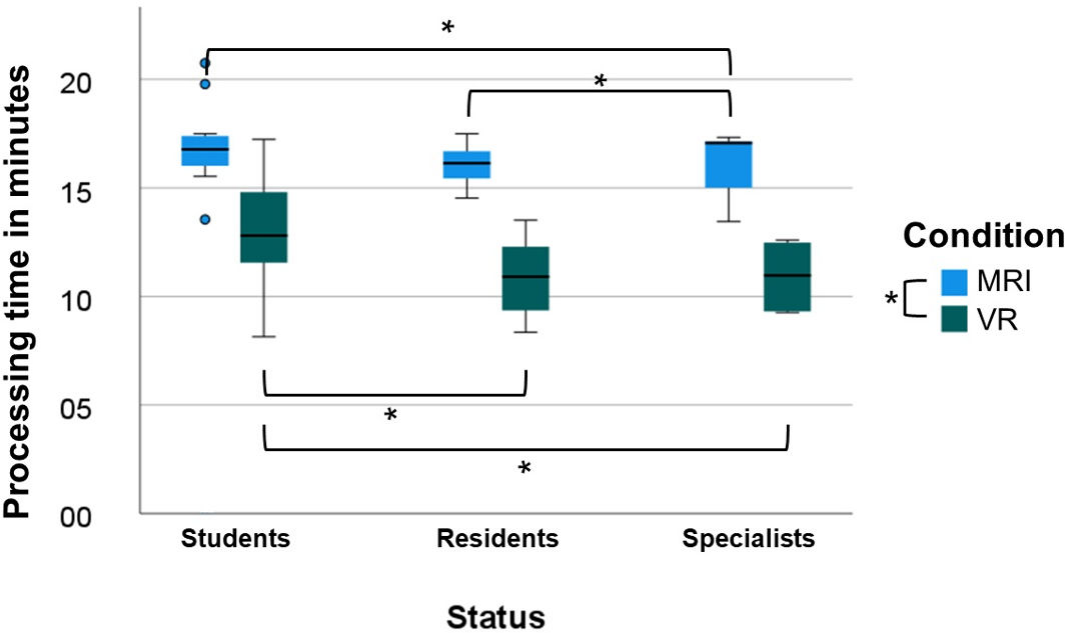
Processing time in minutes; comparing the different conditions (blue=MRI and green=VR) and contrasting the status (student, resident, and specialist). The box plot shows the median, the 2 quartiles, and the extreme values. The black circled dot indicates an outlier value. MRI: magnetic resonance imaging; VR: virtual reality. *indicates statistical significance between the groups.

The analysis of the processing time showed an average processing time using the MRI diagnostics of 16.25 (SD 1.25) minutes. With the additional use of VR, the average value was 11.45 (SD 2.26) minutes (*P*=.001; Figure 3).

The multifactorial 2-way repeated measures ANOVA showed a significant difference in error rates between the MRI and the VR condition (*F*_1,59_=280.700; *P*<.001) and an effect size of 0.826. Furthermore, a significant interaction between condition and status could be presented (*P*=.04). In the pairwise comparison analysis, there was a significant difference in the processing time using MRI diagnostics between students and specialists (students: median 16.46, IQR 16.04-17.24 minutes and specialists: median 15.12, IQR 14.16-17.04 minutes; *P*=.02) as well as between residents and specialists (residents: median 16.54, IQR 15.43-17.27 minutes and specialists: median 15.12, 14.16-17.04 minutes; *P*=.047). No significant results emerged between residents and students (*P*=.33).

Using VR, significant effects emerged between students and residents (students: median 12.44, IQR 11.25-14.42 minutes and residents: median 10.52, IQR 09.21-12.00 minutes; *P*=.004) and students and specialists (students: median 12.44, IQR 11.25-14.42 minutes and specialists: median 09.19, IQR 08.36-12.32 minutes; *P*=.02). No significant differences were found between residents and specialists in the analysis (*P*=.72).

### System Usability

The analysis of the VR system usability was assessed by an adapted SUS. The analysis was carried out based on group. The general acceptance of the system was good in every dimension of the adapted SUS in all groups (Table 2; Multimedia Appendix 5).

With regard to the questions about benefits and problems with both conditions, all groups gave similar answers, which were in favor of the VR condition (Table 3).

**Table 2.** Answers to the questions about general problems and benefits of the virtual reality conditions.^a^

Question	Students, mode (range)	Residents, mode (range)	Specialists, mode (range)
Fun factor	7 (5-7)	7 (6-7)	7 (4-7)
Amount of learning needed before using	1 (1-5)	1 (1-2)	2 (1-5)
Feeling safe using	5 (2-7)	6 (3-7)	6 (4-7)
Cumbersome handling	2 (1-3)	1 (1-2)	2 (1-5)
Quick to learn technique	7 (3-7)	7 (3-7)	5 (5-7)
Easy to use	6 (4-7)	7 (5-7)	6 (4-7)
Unnecessary complexity	2 (1-4)	1 (1-3)	2 (1-3)
Option of regular use	7 (3-7)	6 (5-7)	6 (5-7)

a The values for each question and each group are on a scale of 1=completely disagree to 7=completely agree.

**Table 3.** Answers to the questions about general problems and benefits of the VR^a^ and MRI^b^ conditions.^c^

Question	Students, mode (range)	Residents, mode (range)	Specialists, mode (range)
VR can be a useful additional diagnostic tool	7 (5-7)	7 (6-7)	7 (4-7)
VR is too complex for everyday clinical use	2 (1-5)	2 (1-6)	2 (2-6)
VR can be a beneficial teaching tool	7 (5-7)	7 (6-7)	7 (5-7)
VR helped to better understand the topographical conditions	7 (5-7)	7 (6-7)	6 (5-7)
With VR, I was able to answer the questions with certainty	6 (3-7)	5 (5-7)	6 (1-7)
With MRI, I was able to answer the questions with certainty	2 (1-6)	2 (1-5)	2 (1-5)

a VR: virtual reality.

b MRI: magnetic resonance imaging.

c The values for each question and each group are on a scale of 1=completely disagree to 7=completely agree.

## Discussion

### Findings, Objection, and Comparison to Prior Work

Our study focused on the usability of an additional use of 3D technology in a VR simulation for medical diagnostics to improve the visualization of anatomical structures and pathological findings. We assessed the usefulness of the VR technology as an additional tool in the medical field through an interindividual comparison. Participants were grouped according to their level of medical training and answered 25 MC questions first using sectional imaging (MRI) in a 2D environment (computer screen) and afterward with the respective segmented 3D model in a VR simulation. The main criteria were the number of correctly answered questions and the processing time. Secondary analyses were regarding the usability of VR technology using the SUS. The analysis showed that with the additional use of VR, there was a significant improvement in both main criteria as well as in the subjective perception that the questions were easier to answer in VR.

These results are especially important in light of the essential aspects in medicine, which include the knowledge of anatomical structures, the recognition of pathologies, the establishment of an appropriate diagnosis, and the indication of therapies [2-4,25]. Imaging procedures often play a decisive role in this context. In particular, the evaluation of MRI images is not always easy, as a cognitive transfer from a sectional image that is normally viewed in a 2D environment must be extended to a 3D environment. This mental transformation often correlates with the user’s experience [2,3,22,25-27]. This inductive study was intended to examine the potentials of VR technology in the assessment of liver metastases compared to MRI across different levels of medical experience.

Our study demonstrated that the additional use of VR resulted in a significant improvement in the number of correctly answered MC questions and a reduction in the error rate across all groups. There was an average improvement of approximately 85% compared to using MRI alone.

Students performed significantly worse in answering the questions using MRI alone compared to residents and specialists. However, with the additional use of VR technology, this difference can no longer be shown. Thus, in this study, the student level is raised to that of a resident or a specialist. These results were achieved despite most students had little to no experience with the use of VR technology compared to the residents and specialists. Similar results were shown by Weyhe et al [18]. This can be interpreted as a statement of the quick and easy-to-learn handling as well as an understanding of VR technology, as also described by Schlegel et al [28].

Regarding the processing time, the additional use of VR technology results in a reduction of the average time needed of around 29%. These findings indicate that using VR technology has the potential to facilitate faster anatomical learning [15].

Further detailed analyses showed that both students and residents differ significantly from the group of specialists in the processing time using MRI to answer the questions. However, compared with each other, there is no significant difference between students and residents. This suggest that a high level of prior experience is required for the use of MRI as a diagnostic tool, as is evident in daily clinical practice and also shown by Nasi-Kordhisht et al [29]. In VR, there is a significant difference in the average processing time between students and residents, but no further significant difference between the group of residents and specialists. This seems to reflect the improvement of residents to a specialist level. Although the average processing time in the student group improved, the improvement was not statistically significant. The statistically nonsignificant difference could be attributed, among other things, to the small sample size or by a lack of clinical experience. Residents as well as specialists face the necessity of recognizing and working out the anatomy daily so that a more precise understanding might be created in a shorter time with the additional use of a 3D illustration [2,6,28,30]. Likewise, the students’ nonsignificant improvement may be due to less experience with VR [6]. Furthermore, initial experiences with VR technology can be overwhelming, and to experience it, a new “reality” must first be processed. In addition, there might be a possible playfulness as a student to try new things, which could distract from the actual task. Further, as shown by Walter et al [31,32], there could have been a certain skepticism toward an unknown technology [31,32]. In this case, having experience with VR and being experienced in the field of targeted diagnostics can have a positive effect.

As a secondary objective, all participants were asked about their satisfaction with the use of VR through a well-tested questionnaire, the SUS [23]. Here, a clear endorsement toward the use of VR could be shown. Aside from pure usability, subjective benefits showed up in the areas of simple learning, topographic understanding, and subjective security in answering the questions [15,28,33,34]. Additionally, a high fun factor was indicated [18]. Further, VR is seen as a benefit in clinical teaching as well as a future additional tool for diagnostics [30,33-37]. However, many respondents stated during our study that VR still seems too costly and too elaborate for everyday clinical use.

Regarding VR sickness, our study found minimal relevance. Among a diverse group of participants, which included different sex and age groups, only 1 participant showed mild symptoms most likely caused by VR sickness. This therefore affects about 2% (n=1) of our participants, which is a considerably lower value than reported in comparable studies [38].

The potential integration of VR into everyday clinical practice warrants evaluation. Nowadays, the gold standard in diagnostic imaging continues to be segmental (2D) diagnostics using MRI and CT [5]. Integrating VR would require several changes regarding acquisition costs, premises, integration into everyday life, and ethical and legal issues [39]. Furthermore, there is a need for further improvement in the creation of the virtual 3D representation in terms of time and personnel constraints so that this technology can be integrated into the clinical routine in a simplified way. Finally, the acceptance of the new technology by hospital staff is also pending [31,32,38]. In principle, it seems further basic research is also required in this area.

In our study, the transfer of conservatively learned anatomical knowledge to complex clinical aspects was investigated. It can be concluded from the different results that by using VR, an improvement of the transfer of the learned knowledge to clinical situations is possible. This can be explained by a possible simplification of topographical understanding through additional 3D presentation [16,36,40]. Another reason might be that in VR imaging, certain anatomical details can be displayed significantly better or only seen in VR [36,37]. With enhanced visualization of the anatomical structures, an improved anatomical and topographical understanding occurs, especially with a mismatch between learned anatomy and existing anatomical variations of an individual patient. Here, VR technology is a useful tool to highlight these potential differences in the preparation of an intervention by enhancing the representation of the actual anatomical situation, as shown by Pommert et al [14] and McDonald and Shirk [36]. In addition, Zawy Alsofy et al [41] demonstrated the practical benefits of this enhanced visualization by using VR technology for more accurate surgical planning and comparing it to using conventional imaging alone. Taking into account comparable studies that include cognitive load theory with an impact on the learning process in these studies, it can be stated that the use of VR can reduce the cognitive load and thus may have a positive effect on the result [10,20,21].

In general, these results are difficult to compare due to the few similar studies available. However, the study results suggest that additional 3D technology could have a significant effect on the understanding of anatomical features and could improve clinical diagnostics and planning of treatments and surgeries [15,30,33-37,41,42].

### Limitations

Regarding the limitations of our study, several issues arise. First, the restriction in the comparability with other studies and thus the difficulty of generalizing the data become apparent. The field of VR and clinical practice is explored in many studies; however, most of the studies also have an explorative character. In addition, hardly any study compares different professional groups and in particular the comparison between participants at different stages of medical training and the change in the respective level in relation to their level of training. This is the reason why it is challenging to refer to generally valid data in this study regarding this specific topic.

Second, our study was conducted at a single German university hospital, so the generalizability to other hospitals and other countries cannot be verified with certainty. On the other hand, the fact that all participants are from the same hospital and the same health care system, with a Germany-wide standard, also provides a good basis for data collection. Due to a Germany-wide standard, we believe that the findings can also be transferred to other hospitals in Germany. The international comparison still needs to be investigated further. However, due to similar working conditions with everyday time stress and employees with different levels of education and training as well as the same learning curves for MRI diagnostics, we also expect similar results in an international comparison.

Third, it should also be mentioned that no standardized MC questionnaire was used. Thus, the used tests lacked established values for quality criteria such as reliability and validity. However, based on the significant differences demonstrated between the groups in our study, the questions we used seem to be appropriate to highlight a difference between 2D visualization and VR. Further, the study results show only minor ceiling or floor effects.

Fourth, only certain MRI sequences were provided for answering the MC questions, so that the participant could not access the full potential of MRI diagnostics. This limitation had to be done for time management reasons and reflects a common clinical practice in many medical departments. Nonetheless, the most suitable sequences were selected in cooperation with the department of radiology.

Fifth, the time constraint for answering the questions, especially in MRI, may have led to a reduction in the number of correctly answered questions. Questions not answered in time were scored as incorrect. Increasing the time available could potentially improve the rate of correctly answered questions when using the MRI alone. On the other hand, time pressure is constantly present in clinical practice, making this a test of VR’s practicality.

Sixth, an observed improvement of the participants during VR use can partly be explained by a learning effect. In general, however, the effect presented here is strongly significant, so a sole learning effect cannot be argued as the sole cause. Furthermore, cases from everyday clinical practice were used, which could possibly be remembered by physicians, thus creating an advantage. Nevertheless, if the magnetic resonance images were already known, a significant improvement with the VR condition was still achieved.

Finally, the participant collection shows an imbalanced distribution in the groups; in addition, our design does not include randomization. This influences the results to a certain extent. However, due to the heterogeneous group of participants, especially due to the number of specialists, randomization or grouping was ruled out. The decision to use a cross-over design gave us the opportunity to have an adequate study size with a good database for analyzing the results. Another main reason for not using randomization was to investigate the interindividual comparison. The main aim of this study was not to compare the usefulness of MRI versus VR; we wanted to show how the additional use of VR affects the understanding and use of knowledge for each participant, so we did not randomize. In addition, pretests have shown a higher recognition of cases when VR was used first. Here, participants quickly recognized the associated MRI. Conversely, there was a lower recognition value when MRI was used first. This was also one of the reasons for not using randomization.

### Conclusions

The additional use of VR showed a significant improvement in the outcomes regarding correctly answered questions and general processing time across all groups. When transferred to the clinical routine, the add-on use of VR may enhance diagnostic accuracy. This, among other improvements, can be seen in the groups that already had clinical experience. This is also shown in the processing time, so the use of VR may lead to saving time. Both are components in medicine and in daily clinical routines that are of utmost importance for the adequate care and treatment of patients. In general, we see promising future benefits of 3D and VR technologies regarding anatomical understanding and surgical planning.

## Supplementary material

10.2196/60383 Multimedia Appendix 1 Walkthrough the virtual reality simulation video.

10.2196/60383 Multimedia Appendix 2 Questionnaire 1: multiple-choice questions (English).

10.2196/60383 Multimedia Appendix 3 Raw data system usability scale and raw data magnetic resonance imaging or virtual reality.

10.2196/60383 Multimedia Appendix 4 Questionnaire 2: participant personal data (English).

10.2196/60383 Multimedia Appendix 5 Questionnaire 3: system usability scale (English).

10.2196/60383 Checklist 1 STROBE (Strengthening the Reporting of Observational Studies in Epidemiology) reporting guidelines checklist.

## Abbreviations

CT: computed tomography
MC: multiple-choice
MRI: magnetic resonance imaging
STOBE: Strengthening the Reporting of Observational Studies in Epidemiology
SUS: system usability scale
VR: virtual reality
