# Supplementary material for: Virtual Reality in Clinical Teaching and Diagnostics for Liver Surgery: Prospective Cohort Study
Source: JMIR XR Spat Comput. 2024 Nov 27;1:e60383. doi: 10.2196/60383 (PMC13202505; doi:10.2196/60383)
Supplement: Multimedia Appendix 2 [file xr-v1-e60383-s002.docx]

### Questionnaire 1)

**Case 25)**

1. **How many metastases are present in the liver shown?**

a) There are no metastases

b) There is 1 metastasis in the liver shown.

c) There are 2 metastases in the liver shown.

d) There are 3 metastases in the liver shown.

e) I cannot answer this question.

1. **In which liver segment is there no metastasis?**

a) Segment 3

b) Segment 1

c) Segment 8

d) Segment 7

e) I cannot answer this question.

1. **Which of these statements is most likely to be true?**

a) The main tumour mass is located ventral to the portal vein bifurcation.

b) The right outlet of the portal vein (R. dexter v. portae hepatis) touches the main tumour mass.

c) The bifurcation of the portal vein is completely encircled by the main tumour mass.

d) The main tumour mass lies in the course of the R. sinister of the V. portae hepatis (left outlet of the portal vein)

e) I cannot answer this question.

1. **Which of the statements is most likely to be true?**

a) Each of the hepatic veins (V. hepatica dextra, V. hepatica intermedia, V. hepatica sinistra) originate separately from the inferior vena cava.

b) The main tumour mass is located between the outlets of the V. hepatica sinistra and V. hepatica intermedia.

c) The main tumour mass is located in the course of the V. hepatica sinistra.

d) None of the answers given

e) I cannot answer this question.

1. **5) Which statement is most likely to be true?**

a) There is a long-distance contact between the inferior vena cava and the main tumour mass.

b) There is an infiltration of the main tumour mass into the inferior vena cava cranial to the hepatic vein outlets.

c) There is no contact between the inferior vena cava and the main tumour mass.

d) None of the above statements apply.

e) I cannot answer this question.

**Case 28)**

1. **How many metastases are there in the liver shown?**

a) There are no metastases

b) There is 1 metastasis in the liver shown.

c) There are 2 metastases in the liver shown.

d) There are 3 metastases in the liver shown.

e) I cannot answer this question.

1. **In which liver segment is there a metastasis?**

a) Segment 5

b) Segment 6

c) Segment 7

d) Segment 8

e) I cannot answer this question.

1. **Which statement is most likely to be true?**

a) The bifurcation of the portal vein is in direct contact with the tumour mass.

b) The main tumour mass lies caudal to the portal vein bifurcation.

c) The right branch of the portal vein (R. dexter v. portae hepatis) is fully surrounded by the metastasis.

d) None of the above answers apply

e) I cannot answer this question.

1. **Which statement is most likely to be true?**

a) The main branch of the hepatic dextral vein is in contact with a metastasis.

b) The V. hepatica intermedia is in contact with a metastasis.

c) The main branch of the V. hepatica sinistra shows a long-distance contact with a metastasis.

d) None of the above answers apply.

e) I cannot answer this question.

1. **Which statement is most likely to be true?**

a) The hepatic vein dextra and sinistra arise from the same origin at the inferior vena cava.

b) The inferior vena cava has a long ventral contact with a metastasis.

c) The inferior vena cava is surrounded medially semi circularly by a metastasis.

d) None of the above answers apply.

e) I cannot answer this question.

**Case 29)**

1. **How many metastases are most likely to be found in the liver shown?**

a) There are no metastases

b) There are 1-2 metastases in the liver shown.

c) There are 3-4 metastases in the liver shown.

d) There are >4 metastases in the liver shown.

e) I cannot answer this question.

1. **In which liver segment is there most likely to be no metastasis?**

a) Segment 2

b) Segment 4

c) Segment 8

d) Segment 7

e) I cannot answer this question.

1. **Which statement is most likely to be true?**

a) The bifurcation of the portal vein lies freely and without contact to a metastasis.

b) The main tumour mass is in contact with the bifurcation of the portal vein from the dorsal side.

c) The R. sinister of the V. portae hepatis is infiltrated by a metastasis.

d) None of the above answers apply.

e) I cannot answer this question.

1. **Which statement is most likely to be true?**

a) The V. hepatica intermedia is surrounded semi circularly by a metastasis.

b) There is an infiltration of the V. hepatica sinistra.

c) The V. hepatica dextra is in contact with the main tumour mass.

d) None of the above statements apply.

e) I cannot answer this question.

1. **Which statement is most likely to be true?**

a) The inferior vena cava is infiltrated medially by a metastasis.

b) The inferior vena cava is infiltrated ventrally by a metastasis.

c) There is no infiltration of the inferior vena cava.

d) None of the above statements apply.

e) I cannot answer this question.

**Case 30)**

1. **How many metastases are most likely to be found in the liver shown?**

a) There are no metastases

b) There are 2-3 metastases in the liver shown.

c) There are 5-6 metastases in the liver shown.

d) There are >6 metastases in the liver shown.

e) I cannot answer this question.

1. **In which liver segment is there most likely to be no metastasis?**

a) Segment 3

b) Segment 6

c) Segment 8

d) Segment 7

e) I cannot answer this question.

1. **Which statement is most likely to be true?**

a) The bifurcation of the portal vein is free of infiltration.

b) The R. dexter of the V. portae hepatis is infiltrated by a metastasis at the main branch.

c) The R. sinister of the V. portae hepatis is infiltrated by a metastasis on the main branch.

d) None of the above answers apply.

e) I cannot answer this question.

1. **Which statement is most likely to be true?**

a) There is an infiltration at the origin of the V. hepatica sinistra.

b) The hepatic dextral vein is semi circularly surrounded by a metastasis.

c) There is an infiltration at the origin of the V. hepatica intermedia from the cranial side.

d) None of the above statements apply.

e) I cannot answer this question.

1. **Which of the statements is most likely to be true?**

a) The inferior vena cava is infiltrated ventrally by a metastasis.

b) The inferior vena cava is infiltrated medially by a metastasis.

c) There is no infiltration of the inferior vena cava.

d) None of the above statements apply.

e) I cannot answer this question.

**Case 45)**

1. **How many metastases are most likely to be found in the liver shown?**

a) There are no metastases

b) There is 1 metastasis in the liver shown.

c) There are 2 metastases in the liver shown.

d) There are 3 metastases in the liver shown.

e) I cannot answer this question.

1. **In which liver segment is a metastasis most likely to be present?**

a) Segment 4

b) Segment 5

c) Segment 8

d) Segment 7

e) I cannot answer this question.

1. **Which statement is most likely to be true?**

a) There is no infiltration into the portal vein bifurcation.

b) The R. dexter of the V. portae hepatis touches a metastasis.

c) The R. sinister of the V. portae hepatis is semi circularly surrounded by a metastasis.

d) None of the answers apply.

e) I cannot answer this question.

1. **Which statement is most likely to be true?**

a) A metastasis lies between the V. hepatis intermedia and the V. hepatica sinistra.

b) The V. hepatis dextra is infiltrated by a metastasis.

c) A metastasis is located dorsal to the inferior vena cava.

d) A metastasis is located on the branch of the dextra hepatic vein.

e) I cannot answer this question.

1. **Which statement is most likely to be true?**

a) The V. hepatica intermedia and the V. hepatica sinistra have the same origin at the V. cava inferior.

b) The V. hepatica intermedia divides into two large branches.

c) The V. hepatica dextra runs medial to a metastasis.

d) The vena cava is in contact with the metastasis.

e) I cannot answer this question.
