# Supplementary material for: Virtual Reality in Clinical Teaching and Diagnostics for Liver Surgery: Prospective Cohort Study
Source: JMIR XR Spat Comput. 2024 Nov 27;1:e60383. doi: 10.2196/60383 (PMC13202505; doi:10.2196/60383)
Supplement: Multimedia Appendix 4 [file xr-v1-e60383-s004.docx]

###
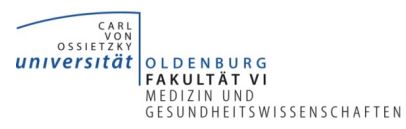
Questionnaire 2)


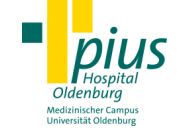


**Pius-Hospital · Georgstr. 12 · 26121 Oldenburg
Department of Visceral Surgery**

**Department director:** Prof Dr. Dirk Weyhe
**Contact:** Joshua Preibisch
Tel.: +49 (0)441 229 1961
Email: preibisch.joshua@gmx.net **Please do not enter a name on this questionnaire**
Address: This study is anonymous!
University hospital – Department of visceral surgery
Georgstr 12
D-26121 Oldenburg

**Title of the study:**

"Benefits of VR technology in clinical teaching and diagnostic imaging as an add-on procedure"

1. Age: __________
2. Gender: __________
3. Which level of education are you at?

O Student

- Year: __________

O Resident

- Year: __________

- Department: __________

O Specialist

- Year: __________

- Department: _________________________________________

- 1. If you are currently studying:

Did you complete a training before studying human medicine (MTA (medical-technical assistant), BTA (biological-technical assistant), CTA (chemical-technical assistant), assistant CTA (chemical-technical assistant), OTA (operating theatre assistant) (operating theatre assistant), nurse or similar?) or similar?

O Yes O No

If yes, please specify type: ____________________________________

1. You are:

O Left-handed O Right-handed

1. Do you own a games console?

O Yes O No

- 1. If yes, how often do you use it?

O 1-2 times a month O 1 time a week O 2-3 times a week O Daily

- 1. Which console(s) do you own? ____________________________

1. Do you already have experience with VR?

O Not used so far O Used 1-5 times O 5-10 times O >10 times

1. How regularly do you use VR?

O No regular use O Regularly monthly O Regularly weekly O Regularly daily
