# Supplementary material for: Virtual Reality in Clinical Teaching and Diagnostics for Liver Surgery: Prospective Cohort Study
Source: JMIR XR Spat Comput. 2024 Nov 27;1:e60383. doi: 10.2196/60383 (PMC13202505; doi:10.2196/60383)
Supplement: Multimedia Appendix 5 [file xr-v1-e60383-s005.docx]

### Questionnaire 3)

***System Usability Scale***Please rate the extent to which you agree with the following statements regarding the use of VR simulation.

|  | Strongly disagree | Disagree | Somehow disagree | Neutral | Somehow agree | Agree | Strongly agree |
| --- | --- | --- | --- | --- | --- | --- | --- |
| I can well imagine using VR on a regular basis. | **☐** | **☐** | **☐** | **☐** | **☐** | **☐** | **☐** |
| I find VR unnecessarily complex. | **☐** | **☐** | **☐** | **☐** | **☐** | **☐** | **☐** |
| I find VR easy to use. | **☐** | **☐** | **☐** | **☐** | **☐** | **☐** | **☐** |
| I can imagine that most people quickly learn to master VR. | **☐** | **☐** | **☐** | **☐** | **☐** | **☐** | **☐** |
| I find the operation of VR very complicated. | **☐** | **☐** | **☐** | **☐** | **☐** | **☐** | **☐** |
| I felt very safe using VR. | **☐** | **☐** | **☐** | **☐** | **☐** | **☐** | **☐** |
| I had to learn a lot before I could work with VR. | **☐** | **☐** | **☐** | **☐** | **☐** | **☐** | **☐** |
| I enjoyed working with VR. | **☐** | **☐** | **☐** | **☐** | **☐** | **☐** | **☐** |
| I can imagine that VR is useful as an additional diagnostic tool. | **☐** | **☐** | **☐** | **☐** | **☐** | **☐** | **☐** |
| VR is too complex for everyday clinical use. | **☐** | **☐** | **☐** | **☐** | **☐** | **☐** | **☐** |
| I can imagine that VR is a benefit in teaching. | **☐** | **☐** | **☐** | **☐** | **☐** | **☐** | **☐** |
| The VR simulation helped me to better understand the topographical conditions. | **☐** | **☐** | **☐** | **☐** | **☐** | **☐** | **☐** |
| I was able to answer the questions in the VR situation with confidence | **☐** | **☐** | **☐** | **☐** | **☐** | **☐** | **☐** |
| I was able to answer the questions with confidence using MRI imaging | **☐** | **☐** | **☐** | **☐** | **☐** | **☐** | **☐** |
